# Steel slag as low-cost catalyst for artificial photosynthesis to convert CO_2_ and water into hydrogen and methanol

**DOI:** 10.1038/s41598-022-15554-3

**Published:** 2022-07-05

**Authors:** Caterina Fusco, Michele Casiello, Pasquale Pisani, Antonio Monopoli, Fiorenza Fanelli, Werner Oberhauser, Rosella Attrotto, Angelo Nacci, Lucia D’Accolti

**Affiliations:** 1 CNR-ICCOM-SS Bari, Via Orabona 4, 70125 Bari, Italy; 2 Chemistry Department, University of Bari, Via Orabona 4, 70125 Bari, Italy; 3 CNR-NANOTEC-SS Bari, Via Orabona 4, 70125 Bari, Italy; 4 CNR-ICCOM, Sesto Fiorentino, Firenze Italy; 5 Research and Development Department, Acciaierie d’Italia S.p.A., SS Appia km 648, 74123 Taranto, Italy

**Keywords:** Environmental sciences, Chemistry, Energy science and technology, Materials science

## Abstract

Photoreduction of CO_2_ with sunlight to produce solar fuels, also named artificial photosynthesis, is considered one of the most attractive strategies to face the challenge of reducing greenhouse gases and achieving climate neutrality. Following an approach in line with the principles of the circular economy, the low-cost catalytic system (**1**) based on an industrial by-product such as steel slag was assessed, which was properly modified with nanostructured palladium on its surface in order to make it capable of promoting the conversion of CO_2_ into methanol and hydrogen through a two-stage process of photoreduction and thermal conversion having formic acid as the intermediate. Notably, for the first time in the literature steel slag is used as photoreduction catalyst.

## Introduction

One of the main concerns related to environmental impact of human activity is the carbon dioxide emission, which is responsible for the global warming. In the short term, the current fossil-based technologies which are the primary cause of CO_2_ production, are unlikely to be replaced by more sustainable one. To achieve the goal of climate neutrality by 2050, the World Community has given a strong incentive to renewable energies, but the search for processes aiming at capturing and valorising carbon dioxide still remains of crucial importance.

**Scheme 1:**
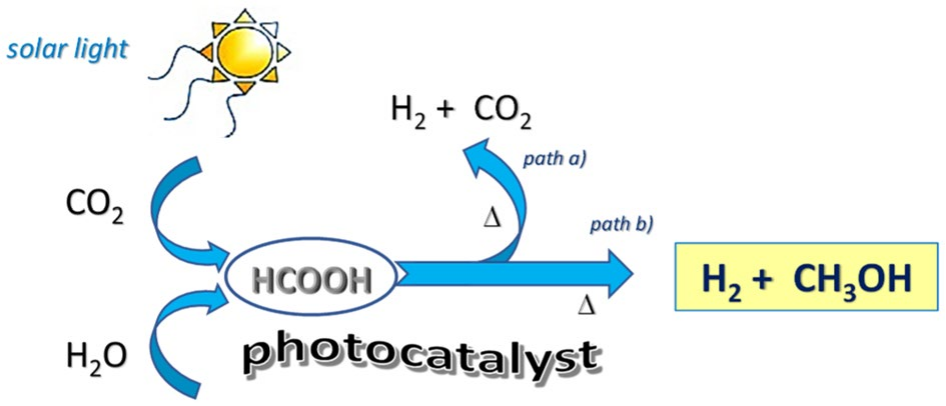
Conversion of CO_2_ and H_2_O in solar fuels.

In this context, photocatalytic reduction of CO_2_ with H_2_O using solar energy, well-known as artificial photosynthesis, is considered one of the most elegant and investigated strategies^1,2^. As a result, solar fuels and high-value chemicals such as formic acid, methane and methanol can be obtained^3,4^. Of special interest is the production of formic acid, which is not only a valuable chemical (i.e. preservative and antibacterial agent), but also considered a hydrogen storage compound with potential application in fuel cells^5^, being prompt to release H_2_ by thermal decomposition^6^ (Scheme 1, path a). Since CO_2_ is regenerated during this latter pathway, the concentration of the greenhouse gas is not reduced in the overall process.

Hence, it would be useful to convert formic acid into a carbon containing compound, in this respect, up to date only a few methodologies^7,8^, mostly published in 2021, have been developed which make use of a photovoltaic (PV) and electrochemical cell (EC), equipped with a heterogeneous catalyst composed by copper supported onto a low-cost perovskite^7^. In this method, light hydrocarbons C_2_H_4_ and C_2_H_6_ were obtained (40%) as well as CO, formic acid and hydrogen^7^.

Inspired by the Circular Economy principles and following our ongoing interest in developing sustainable synthetic protocols^9,10^, we paid attention to steel slag, an industrial by-product coming from the steelmaking process, which we previously used as catalyst in biodiesel production^11^ and that has never been employed in photocatalysis.

Herein we report on the usage of the slag, which has been decorated with nanostructured-palladium on its surface (*i.e.* Pd@slag, **1**) and successively used in a two-step reaction, which consisted in a two-step reaction of the photocatalytic reduction of CO_2_ and water into formic acid followed by its thermal decomposition into hydrogen and methanol (Scheme 1, path b), the latter being one of the most important chemicals for industry^5,6,12^. Both photoconversion and thermal decomposition occurred with good yields, unprecedent selectivity and under relatively mild conditions.

## Results and discussion

### Characterization of Pd@slag catalyst 1

Pristine steel slag, supplied by AcciaieriedItalia (Italy Taranto Plant), consists of two crystalline phases, namely CaAl_2_O_4_^13^ and Fe_3_O_4_^14^ in a 65 and 35 ratio respectively, based on a Rietveld profile refinement (Supporting Information, Figure S1). Nanostructured palladium was deposited onto the slag by a wet impregnation method^15^ affording the nanocomposite Pd@slag labelled as catalyst **1**.

**Figure 1:**
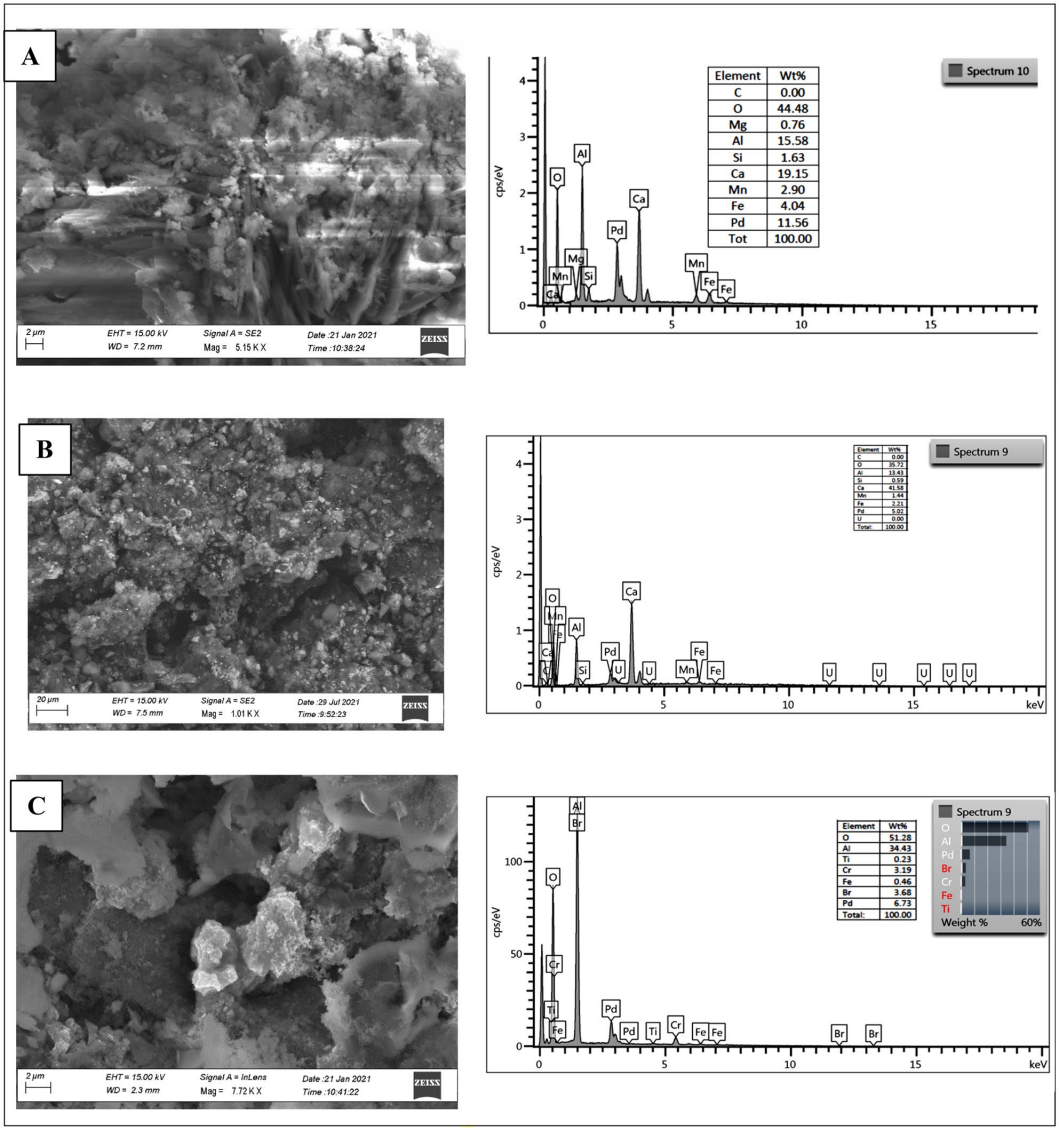
SEM EDX analyses of Pd@slag, 1: (**A**) as-synthesized, (**B**) after photoreduction of CO_2_ and (**C**) after the thermal reaction.

Scanning electron microscopy-energy dispersive X-ray analysis (SEM–EDX) analysis of as-synthesized **1** showed a Pd and Fe content of 12% and 4.04%, respectively (Fig. 1A). Besides, as previously reported^11^, Manganese (2.9%), Calcium, Aluminium, and Oxygen were also detected by EDX analysis, Furthermore, the FT-IR spectrum showed signals at 1636 and 1453 cm^−1^ due to presence of CaO and Fe(II)/Fe(III) oxides (Supporting Information Fig. S2).

**Figure 2:**
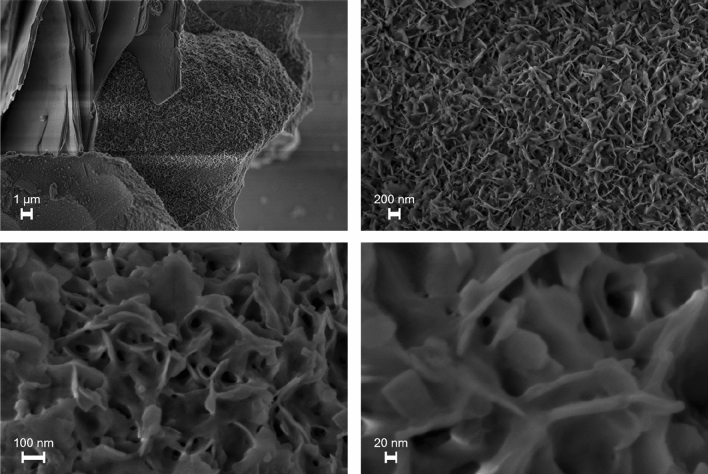
FESEM images of the 1 at different magnifications.

In particular, field-emission scanning electron microscopy (FESEM) images, acquired for **1** proved the presence of metallic Pd with a petal-shaped structure, with a 10 to 20 nm thickness, covering the slag surface (Fig. 2) (see also the Fig. S3. FESEM images of the pristine steel slag in Supporting Information).

**Figure 3:**
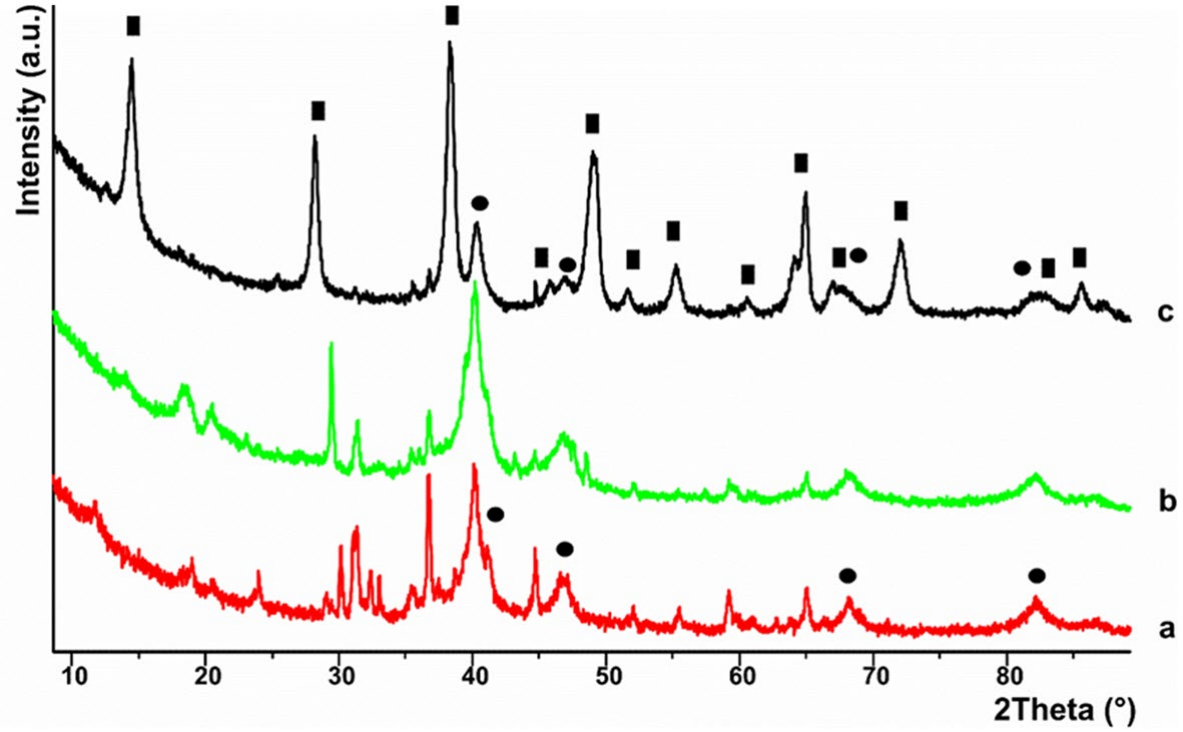
PXRD spectra acquired at room temperature for (1): (**a**) as-synthesized, (**b**) after photochemical reaction and (**c**) after thermal reaction. Full rectangles and circles are assigned to the Bragg reflexes belonging to AlO(OH) and fcc Pd, respectively.

In accordance to the FESEM images, the corresponding XRD spectrum, acquired at room temperature (Fig. 3, trace a) showed the characteristic Bragg reflexes for face-centered-cubic (*fcc*) Pd^16^ (i.e. Pd(111), Pd(002), Pd(022) and Pd(113) at 41.0, 46.0, 68.4 and 82.3° (2Θ), respectively, which were rather intense, due to the high Pd loading, while the Bragg reflexes stemming from support material are depressed, due to the notable coverage of the support surface by Palladium petals.

### UV–Vis studies

UV–Vis DR, which is known to be an important technique to unveil electronic transitions of some metallic species^17^, helped demonstrating that calcium aluminate with iron oxide impurities can act as a photocatalyst.

**Figure 4:**
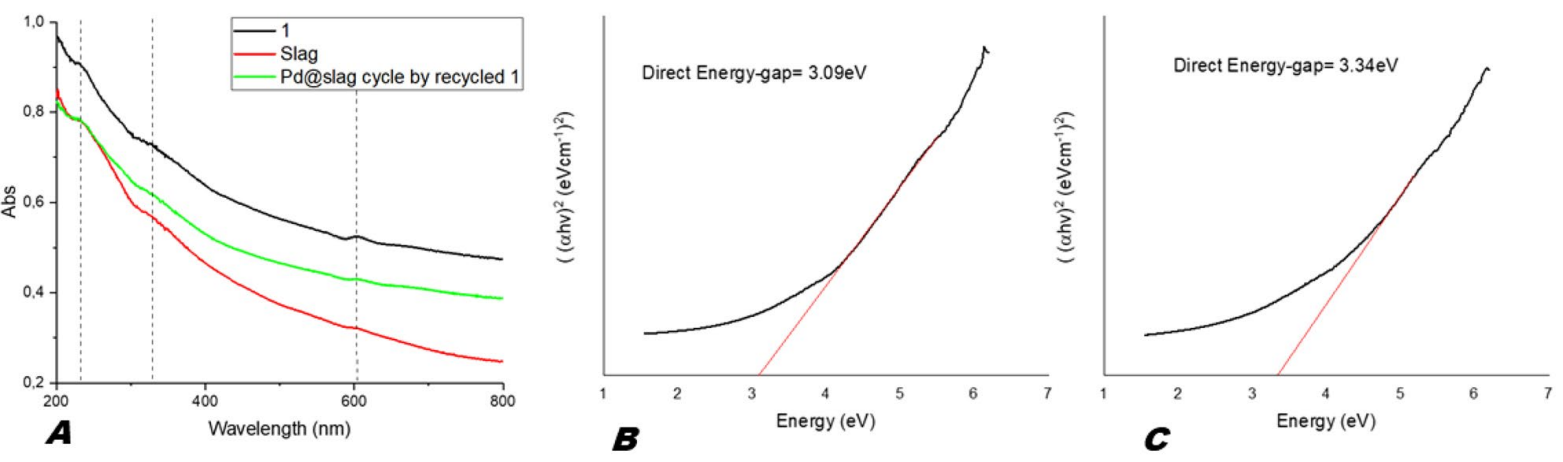
(**A**) UV–Vis DR spectra of slag and catalyst 1, (**B**) Energy gap for slag, (**C**) Energy gap for catalyst 1.

As shown in Fig. 4A the steel slag and **1** had a similar DR spectrum with a peak around to 230–250 nm due to the charge transfer (CT) transition from the ligand, O^2−^ of CaAl_2_O_4_, to Fe^3+^ of the isolated Fe-oxide species, a peak at around 330–380 nm due to the presence of small cluster Fe-oxide particles interacting with the Al ions in the catalyst, and the peak around 610 nm ascribed to Fe^2+^ oxide^17–19^. (see for more details the deconvolution spectra in Supporting information S4*–*6).

The UV–Vis DR could be used to determinate the electronic state of palladium in the catalyst. It has been reported that Pd(II) species present bands at 300–320, 360–370 nm (charge transfer bands) and 440–480 nm (d–d transitions), while highly dispersed PdO particles are characterized by signals at 250–260 nm and 410–420 nm^20^. However, in our case, signals in the range 250–260 nm and 300–370 nm are covered by peaks stemming from slag, while we did not detect any other signal ascribable to Pd(II) such as PdO (around 400–480 nm) or Pd(AcO), precursor of Pd(0) in our case (see also the Fig. S7 in supporting Information). In addition, we did not observe absorptions in the range 520–540 nm and at 650 nm that correspond to charge transfer bands of Pd(I)^20^. On the other hand, Pd(0) is characterized by a continuous absorption without a defined structure in the range 200–800 nm^20,21^. The absence of absorptions in the spectrum of **1**, ascribable to Pd(II) species might confirm the presence of only nano-structured Pd(0) on the support surface, which has been indeed confirmed by PXRD carried out on as-synthesized **1** (Fig. 3, trace a)^20^. Furthermore, the enhanced visible light adsorption obtained for **1** compared to the pristine slag (Fig. 4A)^21,22^ might explain the higher reactivity of the former in the photoreduction of CO_2_.

The band-gap energy has been determined by the Tauc method (*i.e*. optical absorbance data are used to calculate the changes in the band structure. The optic absorption coefficient (*α*) is one of the important parameters that depends on the band structure and the ability of a material to absorb light at a certain wavelength. *α* is given as follows:

$$ \alpha = \frac{{\left( {2.303} \right)A}}{d} $$

where *A* is the absorbance and *d* is the sample thickness.

The absorption coefficient is described by the Tauc relation:coefficient is described by the Tauc re

$$ \alpha h\nu  = B(h\nu  - E_{g} )^{m}, $$

where (*hν*) is the photon energy, *B* is a constant and *E*_*g*_ is the optical band gap between the valence and the conduction band and *m* depends on the type of electron transition (i.e. *m* = *1/2* defines direct allowed transitions and *m* = *2* indirect allowed transitions^23^.

Using this approach we also determined the direct Eg for the steel slag 3.09 Ev (Fig. 4B), while an energy shift to 3.34 eV has been observed for **1** (Fig. 4C), which is in accordance with literature data concerning Fe-Pd interactions^22^, and results obtained for CaAl_2_O_4_ (*i.e*. Eg around 5 eV)^24^.

Finally, the effect of nano-sized Pd on the enhancement of visible light adsorption^22^ gave a strong hint to the higher reactivity of **1** compared to the pristine slag.

### Photocatalytic experiments

Photoreduction of CO_2_ in water with sunlight offers the opportunity to generate renewable fuels in a high sustainable manner. To extend this approach at an industrial level, reactions must be highly efficient and capable of producing biofuels on a high scale. The use of modified heterogeneous catalysts represents the common approach^3,25^, while other strategies make use of sacrificial agents to replace water as a hydrogen donor, although this limits the up-scale of the process^26^.

Photocatalytic reduction of CO_2_ can be performed by various kinds of materials, mainly inorganic and carbon-based semiconductors, but also metal complexes, supermolecules and their derivatives. Usually, photocatalysis is initiated by a semiconductor that absorbs photons with energy equal to or greater than its band gap (Eg). In this way the electrons are excited from the valence band to the conduction band and give rise to the reduction reaction. Typical semiconductors are based on TiO_2_ doped with metals such as Copper, Palladium to mention a few^3,25,27^.

However, recently even many insulator materials, properly modified with transition metals, have shown to behave as photocatalysts. Several studies have demonstrated that the charge transfer excited state of the isolated metal oxo species [Me^(n−1)+^–O^−^]^∗^ into these insulator materials play a similar role as that of photogenerated electron–hole pairs in the semiconductor^19^.

A representative example in the field of CO_2_ photoreduction is given by zeolites, where catalytic activity was attributed to the presence of Fe(III) impurities^17^. In particular, to [Fe(III)–O^2−^] species that can act as a photoactive center by forming [Fe(II)–O^−^]^*^ excitation state suitable for CO_2_ reduction reaction^17,19^.

Based on these findings, we predicted that the slag, essentially composed by calcium aluminate (an insulator) and Fe_3_O_4_, could also show similar performances. This assumption was also corroborated by a recent report showing the photocatalytic properties displayed by Fe_3_O_4_ doped calcium aluminate nanoparticles in degradation processes^28^.

Moreover, due to analogous photocatalytic activity showed by nano-structured palladium^2,29^, we expected that iron impurities combined with nano-structured Pd(0) decorating the slag surface, would confer an enhanced photocatalytic performance to the slag. Likewise, the addition of Pd would enable the successive conversion of the formic acid intermediate into H_2_ and methanol, a process which is known to require Pd(0) as catalyst.

Initially, the two steps of CO_2_ conversion were evaluated separately. Photoreduction was conducted in a sealed photoreactor charged with 20 mL of a suspension of catalyst in bi-distilled water saturated with CO_2_ (Table 1). Reaction conditions in terms of catalyst amounts and irradiation times were chosen based on our previous works^30^.

**Table 1:** CO_2_ photoreduction reactions catalysed by 1.

Entry	Catalyst	Irradiation source^a^	HCOOH^b^ μmol g^−1^ h^−1b^
1	Slag	Sanolux (UV/Vis) Halogen (Vis)	480
2	**1**	Sanolux (UV/Vis) Halogen (Vis)	540
3	**1**	Halogen (Vis)	120
4	**1**	Sanolux (UV/Vis)	18
5	**1**	–^c^	0
6	Lit.^d^		304

General reaction conditions: 30 mg of catalysts in water suspension volume = 20 mL, T = 25 °C, irradiation time = 5 h (see experimental section).

^a^The characteristics of irradiation source were reported in supporting information.

^b^Yields of formic acid were determined by a calibration curve (see supporting information Fig. S10). ^c^Without irradiation (blank reaction).

^d^Eu-MOF as catalyst^5^.

Notably, formic acid was found to be the only one product, as revealed by gas-phase analyses of the head space reactor. Neither H_2_ nor CO were detected.

Interestingly, slag matrix showed a fairly good photocatalytic activity affording 480 μmol g^−1^ h^−1^ of formic acid under simultaneous irradiation of the two radiation sources (Table 1, entry 1).

Under the same conditions, catalyst **1** showed an increased activity (540 μmol g^−1^ h^−1^) due to the presence of Pd nanostructured (Table 1, entry 2).

**Figure 5:**
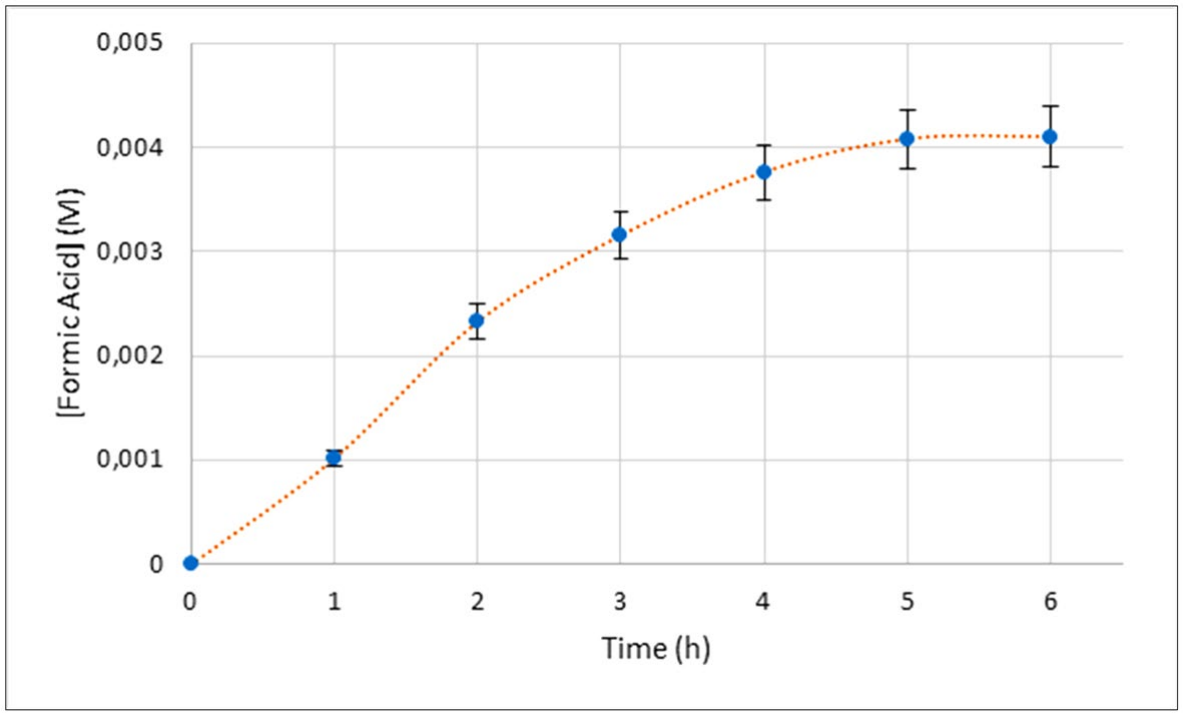
Dotted lines diagram showing the photoreduction trend of 1 during time.

To confirm the latter data, a dotted line diagram was reported for each hour during photoreduction reaction, that showed the increase of the concentration of formic acid in 5 h until reaching a plateau at 4 × 10^−3^ M (Fig. 5).

The evaluation of the influence of irradiation source on the catalyst performance showed that Halogen(Vis) irradiation gives much better results than UV/Vis (Sanolux) (Table 1, entries 3–4). In addition, blank reaction carried out in the presence of **1** without irradiation gave no product (Table 1, entry 5). Notably, photocatalytic activity and selectivity shown by **1** proved to be significantly higher than those of analogous processes reported in the literature (Table 1, entry 6)^5,26,31,32^.

As above mentioned, photoreduction activity can be attributed to Fe_3_O_4_ impurities of slag with the synergic effect of Pd nano-structured^17,29^.

The acquisition of a PXRD spectrum of **1**, recovered after photochemical reaction (Fig. 3, trace b), showed a change in the ratio between the two crystalline phases of the slag. As a result, the intensity of the Bragg reflexes ascribed to CaAl_2_O_4_ decreased compared to those assigned to Fe_3_O_4_. The Bragg reflexes assigned to *fcc* Pd are still present after photocatalytic reaction and the peak profile for the latter phase is comparable to that of as-synthesized **1** (Fig. 3, trace a), excluding hence significant aggregation of Pd. SEM–EDX analysis of recovered **1** (Fig. 1B) confirmed a morphology similar to that of the as-synthesized one, although the quantities of Pd and Fe found in **1** after photocatalysis were lower (*i.e*. Pd, 5.02% *vs* 11.56% and Fe, 2.21% *vs* 4.04%) (Fig. 1B).

### Thermal catalytic reaction

The second step of thermal decomposition of formic acid was surveyed by measuring the production of H_2_, CO, CO_2_ and methanol from a water solution of formic acid (0.44 M) heated at 250 °C for 6 h, in an autoclave reactor, at the presence of catalytic materials (Table 2).

**Table 2:** Thermal decomposition of formic acid.

Entry	Catalyst	Conv^a^	Gas phase composition (%)			CH_3_OH (Yield%)^a^	TOF^b^
			H_2_	CO	CO_2_		
1	–	70	46	3.5	42	–	–
2	Steel slag	95	39	11	38	–	–
3	**1**	95	32	traces	27	6	9
4	Cu/Al					30	0.03^c^

Reaction conditions: 100 mL of Formic acid 0.44 M, catalyst (as reported in experimental section), T (250 °C), t (6 h).

^a^Conversions and yields were determined by GC analyses using calibration curves.

^b^TOF evaluated as mmol of HCOOH converted/mmol of cath. Pd amounts on surface was determined using EDX elemental analysis.

^c^Reaction conditions: 2.61 HCOOH mmol, 12 mmol Cu/4.4 mmol Al at 300 °C, 9 h^34^.

In the blank reaction, carried out without any catalyst, a 70% of formic acid was converted into syngas (CO and H_2_) as well as CO_2_ (Table 2, entry 1). These experimental results are in accordance with the literature^33^, that shows how the thermal decomposition of formic acid in absence of catalyst proceeds by exploiting the two low-enthalpy pathways shown in Eq. (1) (dehydrogenation reaction) and 2 (dehydration reaction).

1 $$ {\text{HCOOH}} \to {\text{H}}_{2} + {\text{CO}}_{2} $$

2 $$ {\text{HCOOH}} \to {\text{CO}} + {\text{H}}_{{2}} {\text{O}} $$

In the presence of steel slag, formic acid conversion increased to 95% and hydrogen, CO and CO_2_ were observed as products (Table 2, entry 2). In contrast, in the presence of catalyst **1**, methanol appeared as new carbon containing product beside CO_2_, while only trace amounts of CO were detected (Table 2, entry 3). Notably, turnover frequency of **1** resulted two orders of magnitudes higher than that of a reference catalyst (Table 2, entry 4)^34^.

Based on the gaseous products obtained by thermal decomposition of formic acid at 250 °C (mainly H_2_ and CO_2_), the formation of methanol observed in the presence of **1** could be attributed to the presence of palladium on the catalyst surface.

**Scheme 2:**
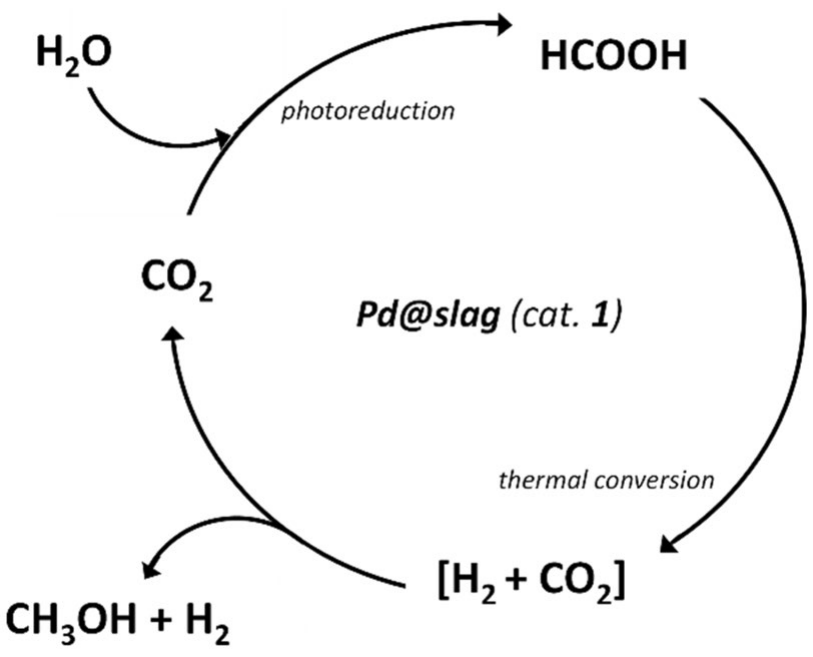
Schematic representation of overall process.

Indeed, it can be assumed that the conversion of HCOOH proceeds through the initial dehydrogenation into H_2_ and CO_2_ (Eq. 1) followed by hydrogenation of this latter to CH_3_OH catalysed by palladium^35^. Of course, only part of HCOOH is converted, as a consequence methanol and hydrogen are the final products besides CO_2_ that virtually can be trapped and recycled (Scheme 2).

However, despite the absence of suitable metals such as Ir and Mo^36^, or of specific reagents^37^, the occurrence of a disproportionation process that leads to methanol, CO_2_ and water cannot be completely excluded.

A PXRD spectrum of **1**, recovered after the thermal reaction (Fig. 3, trace c), showed an almost quantitative conversion of the support material into crystalline Boehmite (AlO(OH))^38^ and only trace amounts of Fe_3_O_4_ are present, whereas the characteristic Bragg reflexes for Pd(0) were clearly present and their shape resembled that of as-synthesized **1** (Fig. 3, trace a) as well as recovered **1**, after photocatalytic reaction (Fig. 3, trace b). Hence the PXRD spectra shown in Fig. 3, proved the stability of Pd(0) against aggregation in the course of the catalytic reactions (*i.e.* photocatalytic and thermal reactions). A SEM image acquired for the recovered catalyst (Fig. 1C) showed the morphological change of the support material after the thermal reaction, confirming hence the results gained by PXRD.

After having validated the efficiency of **1** in the two reaction steps carried out separately, attention was paid to evaluate the feasibility of the overall process performing photochemical and thermal reactions in a consecutive manner. In addition, the robustness of **1** was assessed by carrying out a recycling experiment (Table 3), which proved the feasibility of the overall process promoted by **1** (*i.e.* the second cycle, although the experimental recycling conditions were not optimized, showed a lower formic acid formation and conversion into methanol and hydrogen).

**Table 3:** Recycling experiment of the overall conversion of CO_2_ into methanol and hydrogen.

Cycle	1st step: CO_2_ photoreduction^a^	2nd step: thermal conversion of HCOOH^b^		
	HCOOH (M)^c^	HCOOH Conv. (%)	Products yields(%)	
			CH_3_OH	H_2_
1	2.9 × 10^−3^	98	43	31
2	3.5 × 10^−4^	53	32	21

^a^Reaction conditions: saturated CO_2_-bidistilled water (30 mL), catalyst 1 (45.32 mg), T = 25 °C, irradiation time = 5 h.

^b^Reaction conditions: formic acid suspension of 1st step heated in an autoclave reactor at 250 °C for 6 h.

^c^Conversions and yields determined by GC analyses using calibration curves. CO_2_ by-product (20–40%).

**Figure 6:**
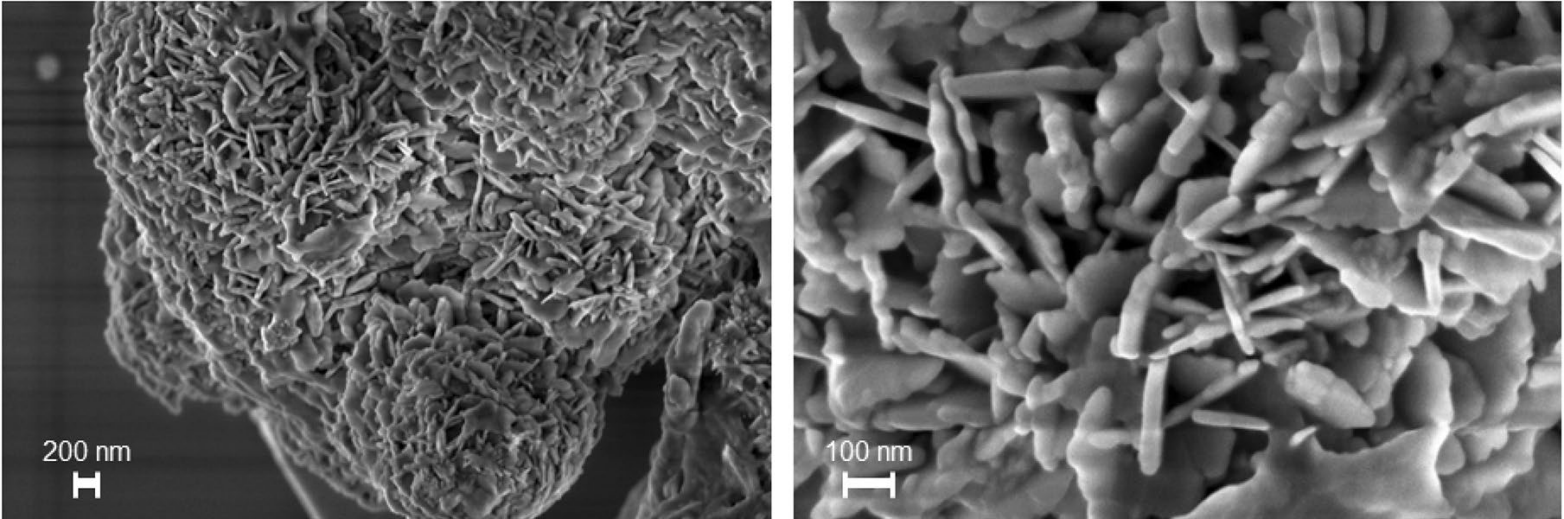
FESEM images of the Pd@slag 1 after one cycle of use in the overall process.

The decrease of the formic acid conversion after the overall cycles might be explained, in accordance with the SEM–EDX analysis conducted for the two separate reaction steps and the SEM–EDX analysis after overall cycle (See supporting information Fig. S8) by the decrease of the Iron and Pd loading during catalysis. Nevertheless, the UV–Vis spectra for **1** and recycled **1** (Fig. 4A) are very similar, confirming thus the presence of the Pd(0) and Fe_3_O_4_ in the recycled catalyst, finally the FESEM image after the overall cycle proved the still presence a petal-shaped structure (Fig. 6).

## Conclusion

In conclusion, these preliminary studies demonstrate that a catalyst material composed by steel slag decorated with Pd structured can convert CO_2_ and water into methanol and hydrogen via formic acid as intermediate, through a two-step reaction involving a photochemical and thermal reaction. Steel slag has been involved for the first time in the photoreduction of CO_2_, most probably by virtue of the iron(III) impurities, while Palladium account for the thermal conversion of formic acid into hydrogen and methanol.

This process is important not only for matching the Circular Economy principles making use of wastes as both reagents (CO_2_) and catalysts (slag), but also for producing in high selectivity an attractive storage medium such as formic acid. In fact, although HCOOH contains only 4.4% hydrogen by mass, it is a precious intermediate because is liquid at room temperature and therefore easy to handle and transport, commercially available on a large scale, and the by-product of H_2_ release, carbon dioxide, can be trapped and either recycled or used as a C1 source for other chemicals. Studies are in progress to ascertain the exact mechanism with which palladium converts formic acid into methanol and hydrogen.

## Experimental

### Materials and method

Solvents and reagents were purchased from Sigma-Aldrich and used as received. Steel slag was a gentle donation of AcciaieriedItalia (Taranto, I). GC/MS analyses were run on a Shimadzu GLC 17-A instrument connected with a Shimadzu QP5050A selective mass detector using a SLB-5MS column (30 m × 0.25 mm id, film thickness 0.25 μm). Mass spectra were performed in EI mode (70 eV). GC/BID analyses were run a on a TRACERA (Shimadzu Italia S.r.l., Milano, Italy); equipped with BID (Barrier Discharge Ionization Detectors) at Helium plasma, using two kinds of columns: for polar compounds HP-Innowax column (30 m × 0.25 mm id, film thickness 0.25 μm). using the following conditions: temp. injection 200 °C, linear velocity 72.6 cm/sec, split ratio: 3, Temperature programm 40°Cx 5 min to 220 °C for 10 min with rate 15 min/sec, to 240 rate 40 min/sec for 10 min, detector 250 °C, for the gas phases Restek widebore column (30 m × 0.25 mm id) using the following conditions: temp. injection 100 °C, linear velocity 54.3 cm/sec, split ratio 0.1, 40°Cx 3 min to 250 °C for 10 min with rate 15 min/sec, for 40 min, to 240 rate 40 min/sec for 10 min, detector 250 °C. ATR-FTIR spectra of catalyst **1** were recorded in the range of 400–4000 cm^−1^ on a Perkin Elmer spectrometer instrument.

Scanning electron microscopy-energy dispersive X-ray analysis (SEM–EDX) analyses were performed with an electron microscope FESEM-EDX Carl Zeiss Sigma 300 VP. The samples were fixed on aluminum stubs and then sputtered with graphite using a Sputter Quorum Q150. Additionally, the chemical composition was determined by EDX under the scanning electron microscope and X-rays diffraction. Field-emission scanning electron microscopy (FESEM) observations of the steel slag and of catalyst **1** powders deposited onto a Si wafer were carried out using a Zeiss SUPRA™ 40 FESEM. FESEM images were acquired at electron acceleration voltage of 2 kV, working distance of 2 mm, magnification up to 500 k × , using an in-lens secondary electron detector.

The powder X-ray diffraction (PXRD) spectra were acquired at room temperature by depositing ground powders of each sample onto a Si wafer (zero background) which was rotated (0.5 Hz) during spectrum acquisition. The spectra were acquired at room temperature with a X’PERT-PRO powder diffractometer, equipped with solid state detector (PIXcel) and a parabolic MPD mirror, using CuKα radiation (λ = 1.54059 Å). The PXRD spectra were recorded in the 2 Theta range 4.00–90° applying a step size of 0.0263° and a counting time of 50.49 s. per step.

Qualitative and quantitative analyses were carried out on a Agilent Cary 5000 spectrophotometer in diffuse reflection (DR) mode. This kind of measurement with a UV–Vis spectrophotometer is used to determine the optical properties of powder materials and consequently the band-gap energy (Eg). An integrating sphere is set as a DR accessory in order to collect the diffused reflection and direct it into a photo detector. The powder sample preparation consists of filling the powder into a cylindrical sample holder and forming a 2 mm thick layer for all incident light to be absorbed or scattered before reaching the back surface of the sample.

The yields of HCOOH, CH_3_OH and H_2_ were determined via GC/BID by means of a calibration curve (see supporting information).

### Preparation of catalyst 1

1.5 g of steel slag (230 mesh) were placed in a round bottomed flask together with 150 mL of water and 630 mg of Pd(OAc)_2_. A solution of NaBH_4_ in methanol, was slowly dropped into the flask under stirring. After all the solution was added, the mixture was stirred to allow palladium nanostructures to form onto the slag surface. After 12 h, the stirring was shut off, the mixture was filtered, and the catalyst 1 was washed with water to remove the excess of NaBH_4_ and dried at 90 °C for 24 h^15^.

### Photocatalysis experiments

The 1st step relating to photocatalytic reactions was performed using a photoreactor equipped with a jacket for the circulation of cooling water and with both HRC UV–Vis 300 W (Sanolux) lamp and a Xe-Halogen 400 W (Radium) lamp placed 18 cm from the reactor^30,39^ (See also supporting information). Reactions were performed using a careful weighted catalyst amount of 0.03 g ca. in 20 mL of double-distilled water. Initially, Argon was bubbled for 1 h to eliminate the presence of air inside, then CO_2_ was bubbled for 1 h to saturate the reaction mixture. Next, depending on the experimental configuration, the lamps were lit up for 5 h^30^. Reactor was sealed during the reaction, the solution was kept under continuous stirring and the temperature inside the reactor was is maintained at 25 °C thanks to the continuous circulation of water in the cooling jacket. At the end of the reaction, sampling of the headspace was carried out by using a 1 mL gas syringe to analyze the gaseous photoreduction products in the vapor phase (CO, H_2_ and starting reagent CO_2_). Subsequently, aqueous solution was analyzed, after centrifugation (ALC CENTRIFUGE PK 110) using GC-BID and GC–MS techniques to determine amount of HCOOH or other photoreduction by-products.

### Thermal experiments

2nd step experiments pertaining the thermal conversion of HCOOH (Table 2) were performed in a stainless-steel reactor by heating, under stirring and at 250° C for 6 h, 100 mL of an aqueous solution of formic acid 0.44 M with suspended 101.71 mg of the Pd@slag catalyst **1**. Generally, pressure inside reactor reached 40 atm measured with a pressure valve. At the end of the reaction, sampling of the headspace was carried out by using a 1 mL gas syringe to analyze the gaseous products in the vapor phase. Subsequently aqueous solution was analyzed, after centrifugation (Alc Centrifuge Pk 110) using GC-BID and GC–MS techniques to determine amount of CH_3_OH or other by-products.

### Overall process procedure and recycling experiments

Both photoreduction and thermal conversion steps were conducted in a sequential manner according to the following procedure (Table 3). In the first step, 30 mL of de-aerated and bidistilled water saturated with CO_2_, were added with 45.32 mg of catalyst (**1**). Suspension was irradiated under stirring at 25 °C for 5 h with Sanolux and Xe-Halogen lamps. At the end of reaction, mixture was placed into the stainless-steel reactor and the second step was started heating under stirring for 6 h at 250 °C. After the first cycle the catalyst was recovered by centrifuging and drying at 60° C for 12 h and reused. The yield for the gas phase was obtained using the calibration curve and the equation PV = nRT at 353 K and 5.92 atm with 0.2 L of constant volume^40^.

## Supplementary Information

Supplementary Information.

## Data Availability

All the Data were reported in the manuscript and Supporting Information.
