# Supplementary material for: Steel slag as low-cost catalyst for artificial photosynthesis to convert CO2 and water into hydrogen and methanol
Source: Sci Rep. 2022 Jul 5;12:11378. doi: 10.1038/s41598-022-15554-3 (PMC9256681; doi:10.1038/s41598-022-15554-3)
Supplement: Supplementary file 1 — Supplementary Information. [file 41598_2022_15554_MOESM1_ESM.pdf]

Supporting Information

for

Steel Slag as Low-Cost Catalyst for Artificial Photosynthesis to convert CO_2 and Water into Hydrogen and Methanol

Caterina Fusco,[1] Michele Casiello,[2] Pasquale Pisani,[2] Antonio Monopoli,[2] Fiorenza Fanelli,[3] Werner Oberhauser,[4] Rosella Attrotto,[5] Angelo Nacci,[1,2] Lucia D'Accolti[1,2] *

[1] CNR-ICCOM-SS Bari via Orabona 4 70125 Bari Italy
[2] Chemistry Department -University of Bari, via Orabona 4, 70125 Bari Italy
[3] CNR-NANOTEC-SS Bari via Orabona 4 70125 Bari Italy
[4] CNR-ICCOM Sesto Fiorentino Firenze Italy
[5] Acciaierie d'Italia S.p.A. Research and Development Department SS Appia km 648 - 74123 Taranto, Italy

(16 pages, including this cover)

Table of Contents

PXRD spectrum of the slag acquired at room temperature……………………………………p.S2
FTIR-ATR spectrum of Pd-slag virgin……………………………………………………...p. S3
FESEM images of the pristine steel slag…………………………………………………p. S4
Deconvolution UV-vis DR spectra………………………………………………………..p. S5-7
UV-vis DR spectrum of Pd(AcO)2………………………………………………………p. S8
SEM-EDX of catalyst after overall first cicle……………………………………………p. S9
HRC UV-VIS lamp 300W (Sanolux) and Xe-Halogen lamp 400W (Radium) spectra…………p.S10
Calibration curve for determination of HCOOH………………………………………..p. S11
Calibration curve for determination of CH_3OH [10^{-5}-10^{-4}M] ……….………………….......p. S12
Calibration curve for determination of CH_3OH [10^{-3}M] ……….…………………….......p. S13
Calibration curve for determination of H_2…….………………………………......p. S14
Calibration curve for determination of CO…….…………………………………………p. S15
Calibration curve for determination of CO_2……………………………………………p. S16

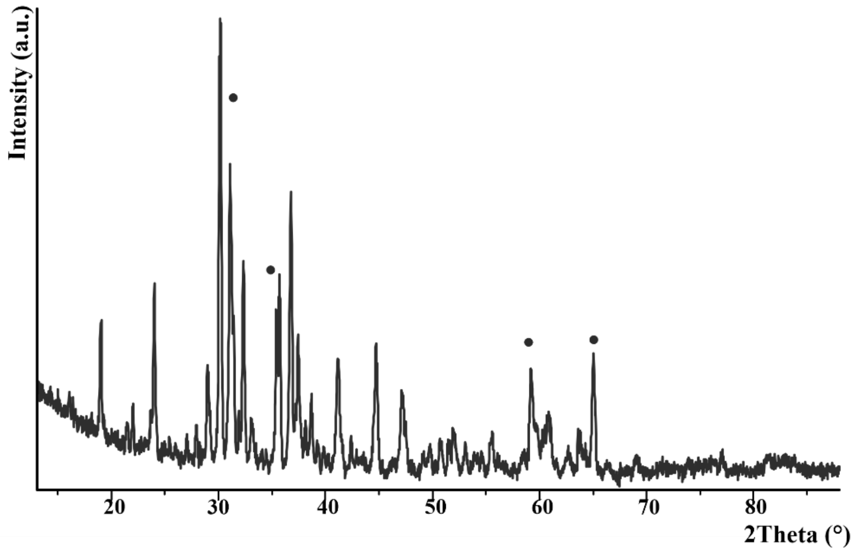

Figure S1. PXRD spectrum of the slag acquired at room temperature: The Bragg reflexes marked with a full circle belong to Fe_3O_4.

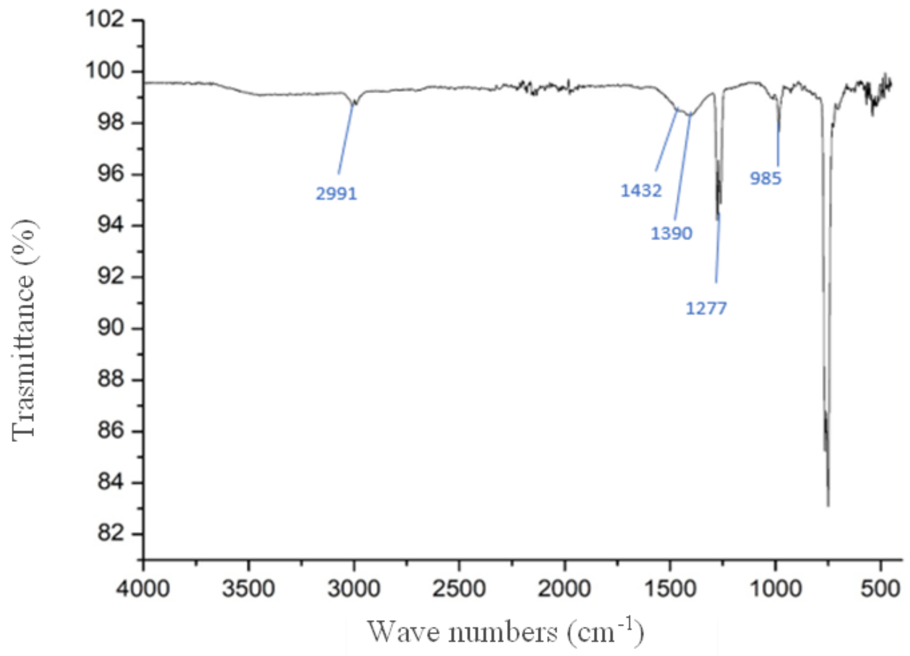

Figure S2. FTIR-ATR of Pd-slag virgin

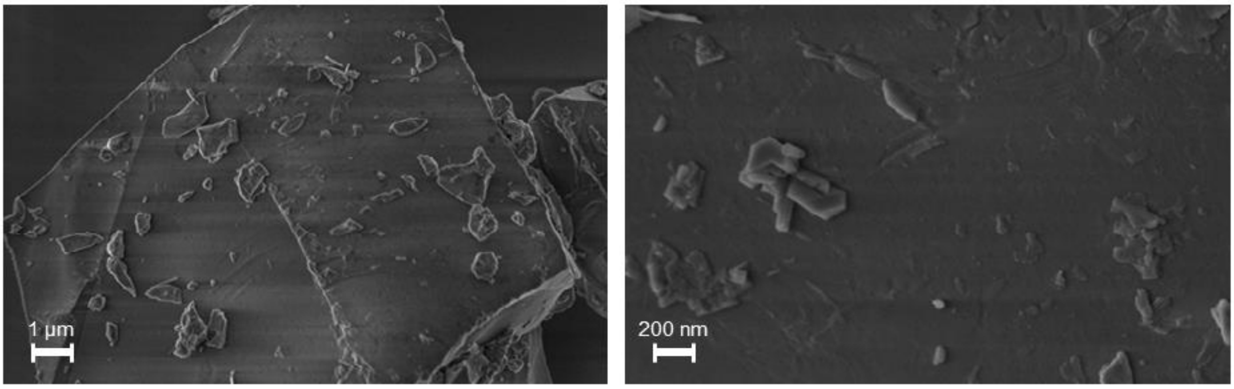

Figure S3. FESEM images of the pristine steel slag

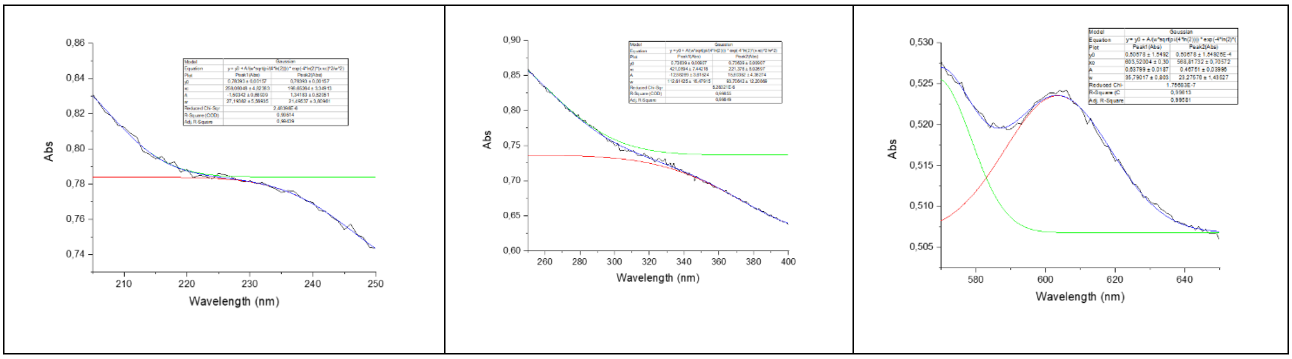

Figure S4. deconvolution UV-vis DR Spectrum of pristine steel slag

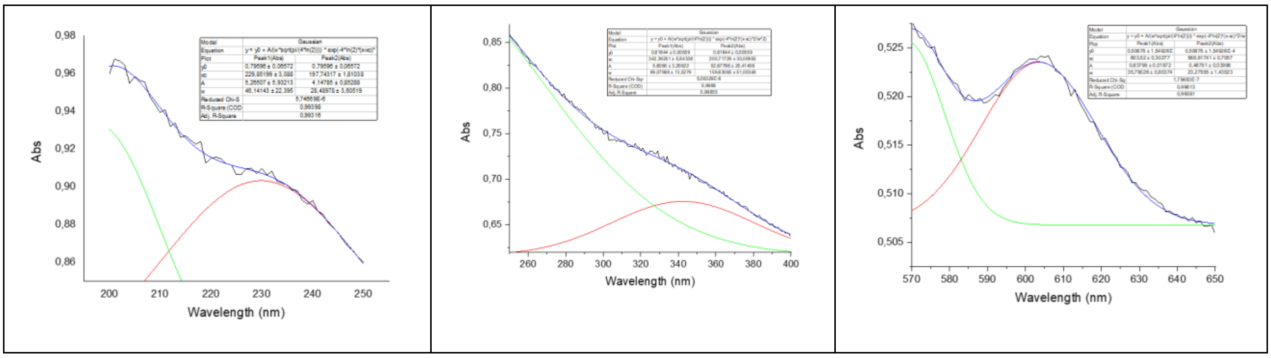

Figure S5. deconvolution UV-vis Spectrum of Pd@slag

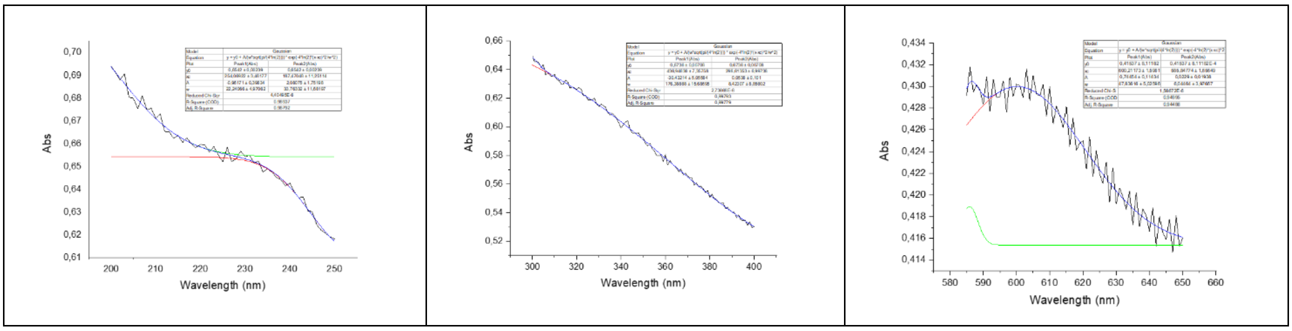

Figure S6. deconvolution Uv-vis spectrum of the catalysts after overall cycles

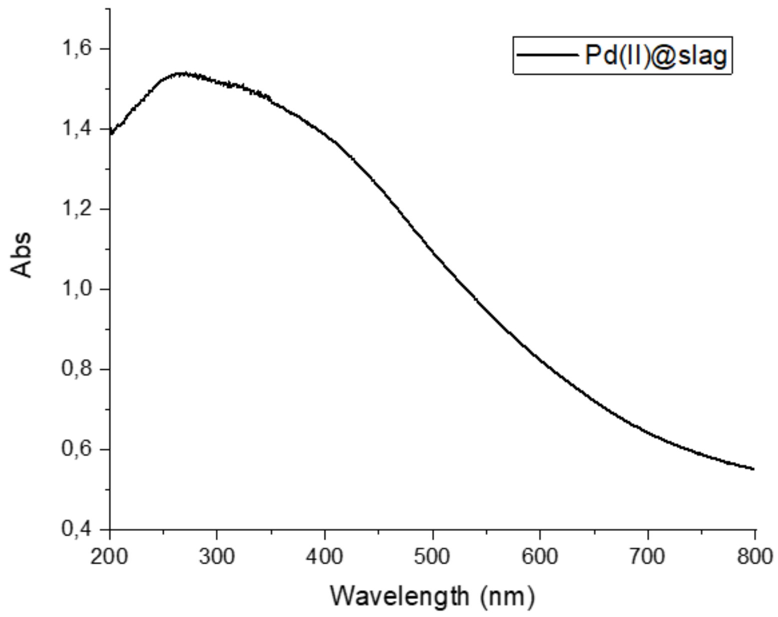

Figure S7. UV-vis DR spectrum slag with impregnate $Pd(OAc)_2$

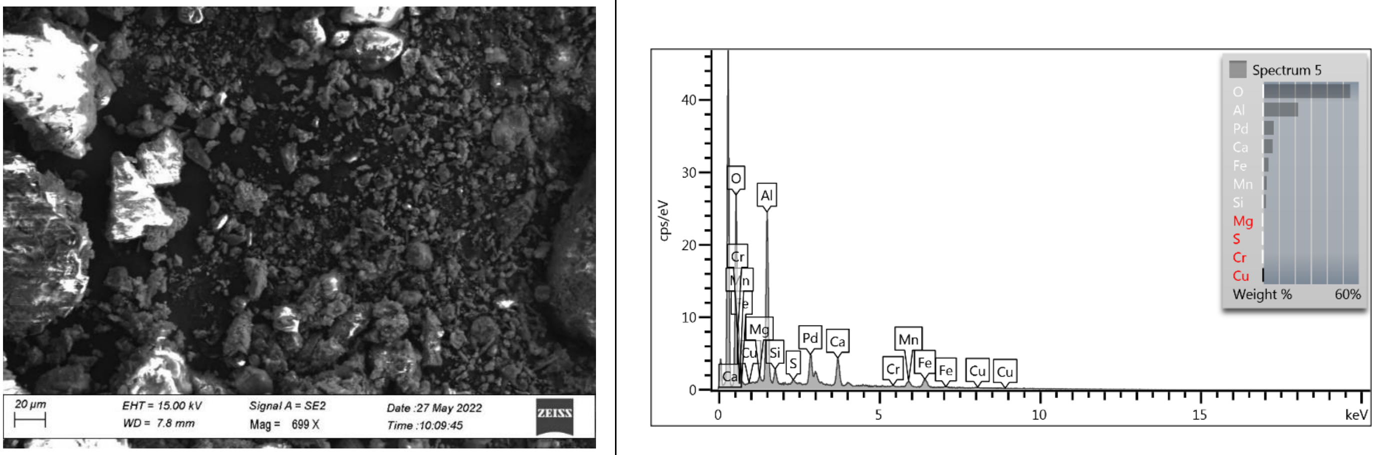

Element	Wt%	Wt% Sigma
O	54.62	0.33
Mg	0.74	0.08
Al	22.09	0.20
Si	2.11	0.09
S	0.63	0.07
Ca	6.20	0.12
Cr	0.40	0.09
Mn	2.47	0.13
Fe	3.70	0.15
Cu	0.02	0.24
Pd	7.01	0.16
Total:	100.00	

Figure S8. SEM-EDX of catalyst after overall first cicle.

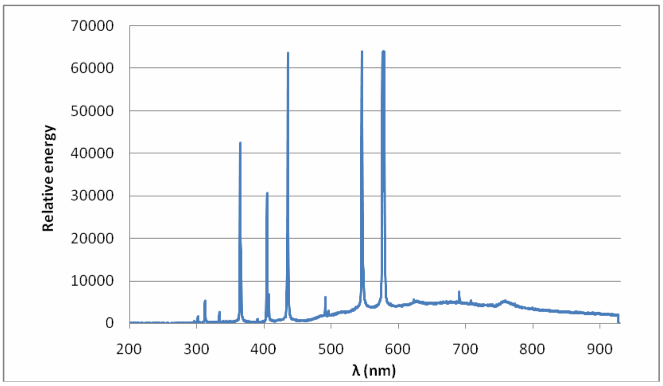

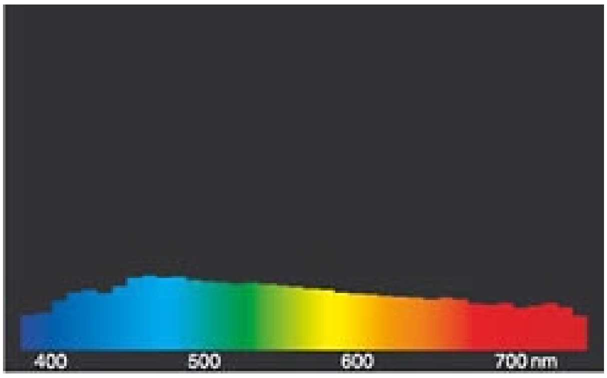

Figure S9. A HRC UV-VIS lamp 300W (Sanolux). (Radium)

Xe-Halogen lamp 400W

Calibration curve for determination of HCOOH

To evaluate yields of HCOOH, a calibration curve was prepared by means of 5 standard solutions of HCOOH into water at different concentration; subsequently the solutions were analysed through gas-chromatograph (Tracera) with a HP-INNOWAX column and with He as a carrier gas; the detector was BID. The experimental parameters are:

HP-INNOWAX column

- Length: 30 m
- I.D.= 0.250 mm
- Film: 0.25 μm
- T. Injector: 200°C

GC-BID:

RATE	T(°C)	HOLD TIME (min)
-	40	5
15	220	5
40	240	10

GC-BID areas were collected (for each solution by means of three replicates and the average area is reported in Fig. S1):

	Concentration (M)	Average area	Standard deviation
1.	0.00020	2270363	102620
2.	0.00040	2435003	37986
3.	0.00081	3029592	93917
4.	0.00161	4384564	144690
5.	0.00242	5861831	93789

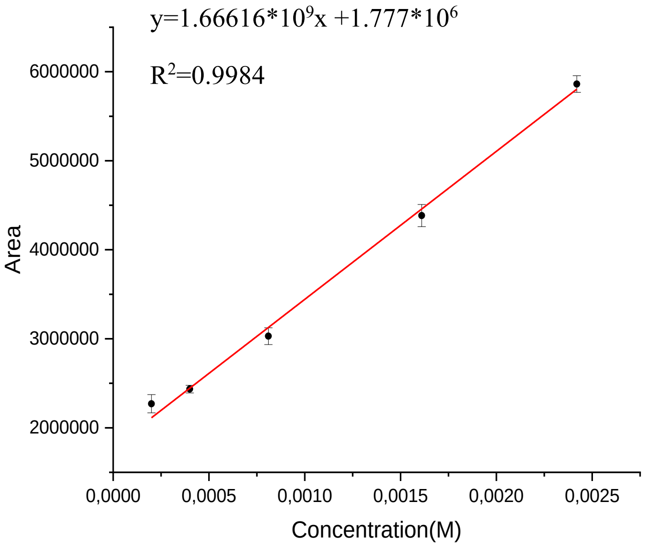

Figure S10. Calibration curve for evaluating yields of formic acid

Calibration curve for determination of CH_3OH [10^{-5}-10^{-4}M]

To evaluate yields of CH_3OH, a calibration curve was prepared by means of 5 standard solutions of CH_3OH into water at different concentration; subsequently the solutions were analysed through gas-chromatograph (Tracera) with a HP-INNOWAX column. The experimental parameters are:

HP-INNOWAX column

- Length: 30 m
- I.D.= 0.250 mm
- Film: 0.25 µm
- T. Injector: 200°C

GC-BID:

RATE	T(°C)	HOLD TIME (min)
-	40	5
15	220	5
40	240	10

GC-BID areas were collected (for each solution by means of three replicates and the average area is reported in Fig.S2):

	Concentration (M)	Average area	Standard deviation
1.	$3*10^{-5}$	1706	402
2.	$4*10^{-5}$	5216	399
3.	$5*10^{-5}$	31116	996
4.	$6.6*10^{-5}$	60618	1618
5.	10^{-4}	170662	1470

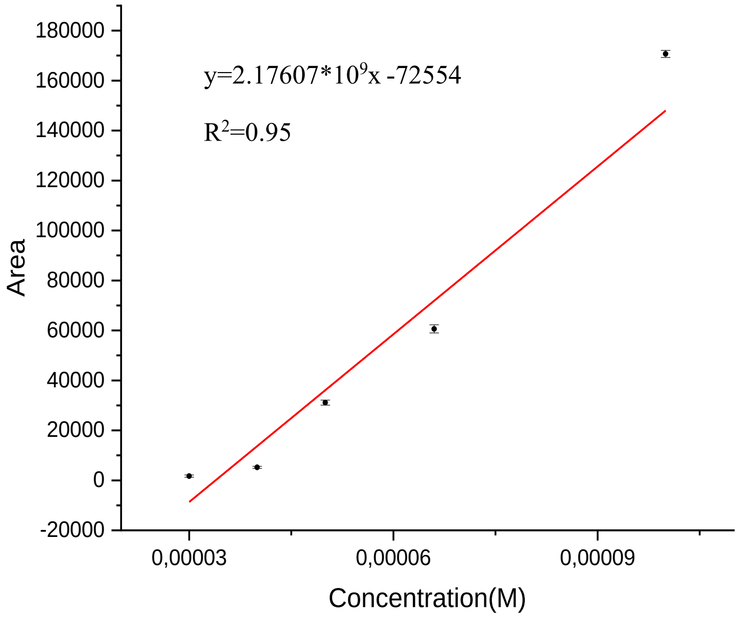

Figure S11. Calibration curve for evaluating yields of CH_3OH

Calibration curve for determination of CH_3OH [10^{-3}M]

To evaluate yields of CH_3OH, a calibration curve was prepared by means of 5 standard solutions of CH_3OH into water at different concentration; subsequently the solutions were analysed through gas-chromatograph (Tracera) with a HP-INNOWAX column. The experimental parameters are:

HP-INNOWAX column

- Length: 30 m
- I.D.= 0.250 mm
- Film: 0.25 µm
- T. Injector: 200°C

GC-BID:

RATE	T(°C)	HOLD TIME (min)
-	40	5
15	220	5
40	240	10

GC-BID areas were collected (for each solution by means of three replicates and the average area is reported in Fig.S3):

	Concentration (M)	Average area	Standard deviation
1.	0.00125	833429	43338
2.	0.00249	930519	21402
3.	0.0049	1116965	69252
4.	0.0065	1295002	16835
5.	0.0098	1534492	35293

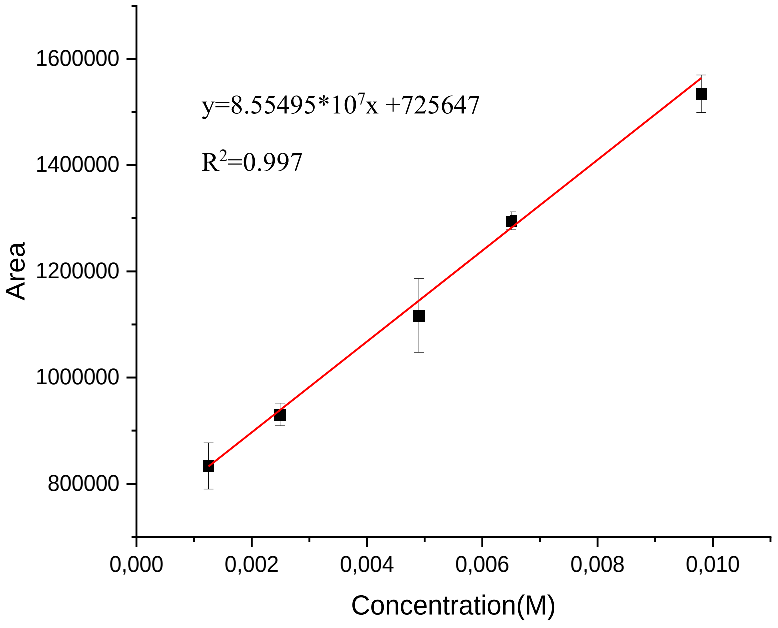

Figure S12. Calibration curve for evaluating yields of CH_3OH

Calibration curve for determination of H_2

To evaluate yields of H_2, a calibration curve was prepared by means of 5 standards of H_2 and air at different concentration directly in gas syringe(1mL); subsequently standards were analysed through gas-chromatograph (Tracera) with a RESTEK column and with He as a carrier gas. The experimental parameters are:

RESTEK column

- Length: 2 m
- I.D.= 0.53 mm
- T. Injector: 150°C

GC-BID:

**Rate**	**T(°C)**	**Hold time (min)**
-	35	2.5
20	250	10
15	270	5.42

GC-BID areas were collected (for each solution by means of three replicates and the average area is reported in Fig. S4):

	**Concentration %**	**Average area**	**Standard deviation**
1.	4	1185313	66377
2.	5	1258154	78005
3.	10	1684871	53916
4.	20	2708870	75848
5.	50	4344378	99920

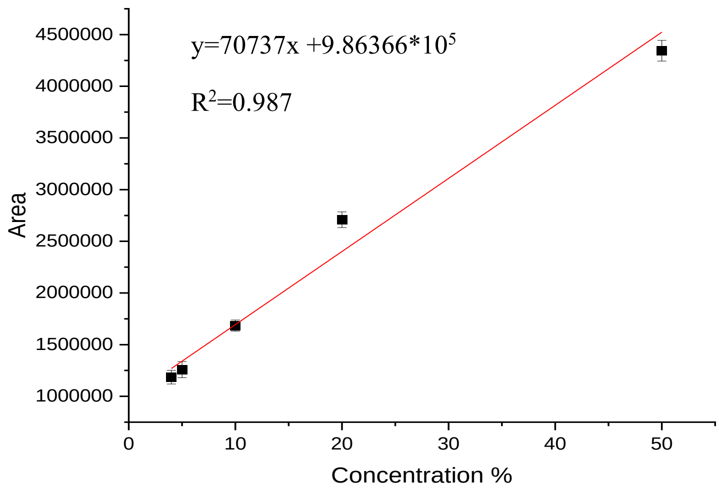

Figure S13. Calibration curve for evaluating yields of H_2

Calibration curve for determination of CO

To evaluate yields of CO, a calibration curve was prepared by means of 7 standards of CO and air at different concentration directly in gas syringe(1mL); subsequently standards were analysed through gas-chromatograph (Tracera) with a RESTEK column and with He as a carrier gas. The experimental parameters are:

RESTEK column

- Length: 2 m
- I.D.= 0.53 mm
- T. Injector: 150°C
-

GC-BID:

**Rate**	**T(°C)**	**Hold time (min)**
-	35	2.5
20	250	10
15	270	5.42

GC-BID areas were collected (for each solution by means of three replicates and the average area is reported in Fig. S5):

	**Concentration %**	**Average area**	**Standard deviation**
1.	4	5717628	285881
2.	5	6105925	360249
3.	10	10520862	347188
4.	20	15700809	455323
5.	30	17358855	563171
6.	40	18028640	272232
7.	50	20222868	469170

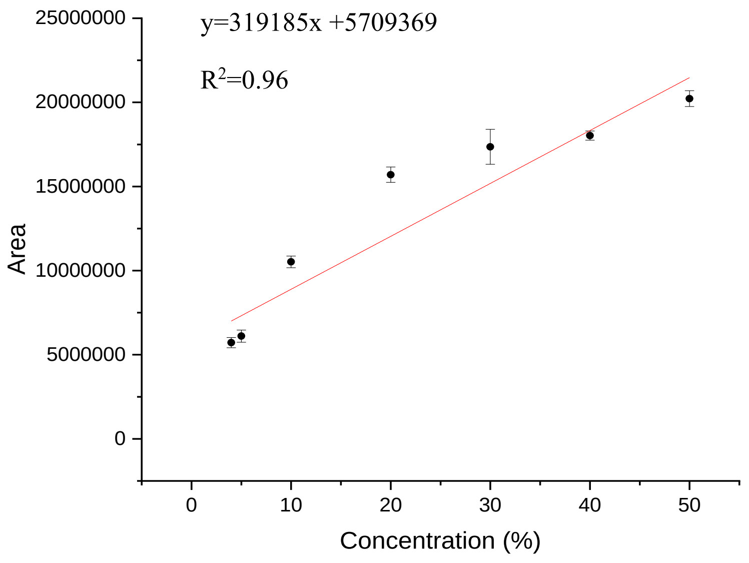

Figure S14. Calibration curve for evaluating yields of CO

Calibration curve for determination of CO_2

To evaluate yields of CO_2, a calibration curve was prepared by means of 4 standards of CO_2 and air at different concentration directly in gas syringe(1mL); subsequently standards were analysed through gas-chromatograph (Tracera) with a RESTEK column and with He as a carrier gas. The experimental parameters are:

RESTEK column

- Length: 2 m
- I.D.= 0.53 mm
- T. Injector: 150°C
-

GC-BID:

**Rate**	**T(°C)**	**Hold time (min)**
-	35	2.5
20	250	10
15	270	5.42

GC-BID areas were collected (for each solution by means of three replicates and the average area is reported in Fig. S6):

	**Concentration %**	**Average area**	**Standard deviation**
1.	1	3321984	428121
2.	10	13774308	1603518
3.	20	19857565	1112034
4.	30	24252599	1964460
5.	40	26915206	942032
6.	50	30860975	2129407

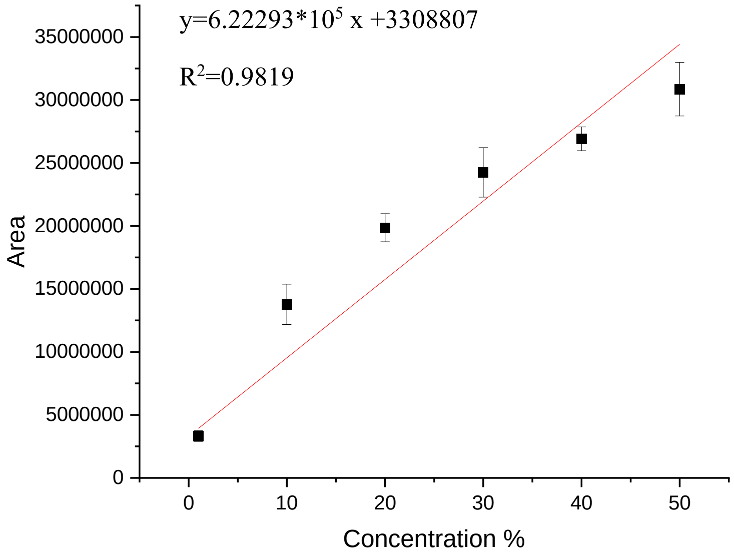

Figure S15. Calibration curve for evaluating yields of CO_2
